# Microvascular anatomy of ovary and oviduct in the adult African Clawed Toad (*Xenopus laevis* DAUDIN, 1802)–Histomorphology and scanning electron microscopy of vascular corrosion casts

**DOI:** 10.1111/ahe.12569

**Published:** 2020-05-24

**Authors:** Alois Lametschwandtner, Bernd Minnich

**Affiliations:** ^1^ Department of Biosciences Vascular and Exercise Biology Research Group University of Salzburg Salzburg Austria

**Keywords:** Histomorphology, ovary, oviduct, scanning electron microscopy, vascular casts, *Xenopus*

## Abstract

Ovaries and oviducts of the adult African Clawed Toad (*Xenopus laevis* DAUDIN, 1802) were studied by light microscopy (LM) of paraplast embedded tissue sections and scanning electron microscopy (SEM) of vascular corrosion casts (VCCs). Histomorphology revealed that ovarian vessels located in the thecal layers. Ovarian and interlobar arteries displayed a horse‐shoe shaped longitudinally running bundle of vascular smooth muscle cells. Follicular blood vessels showed flattened profiles, which were confirmed by scanning electron microscopy in vascular corrosion casts. The flattened profiles obviously led to high intravasal pressures, which locally prevented filling of the follicular capillary bed. Oviduct arteries pierced the fibrous stroma surrounding the oviduct mucosa. In the pars convoluta, the mucosa consisted of a ciliated simple columnar epithelium and tubular oviduct glands that opened between ciliated epithelial cells into the oviduct lumen. Oviduct arteries branched at the basolateral surfaces of tubular glands. After a short tangential course, arterioles branched into capillaries which ran radially between oviduct glands towards the subepithelium. Anastomoses at different heights connected capillaries of neighbouring glands. Subepithelially, capillaries ran longitudinally and undulated. Postcapillary venules radiated centrifugally towards the stroma to finally drain into oviduct veins located in the stroma. Oviduct vascular densities clearly reflected non‐ovulatory and ovulatory states.

## 1 INTRODUCTION

In the last two decades, many studies focused upon endocrine‐disrupting chemicals (EDCs) and their effects on reproduction in animals and men. To study these effects in water‐living amphibians, the African Clawed Toad (*Xenopus laevis* DAUDIN*,* 1802) has become a new model organism (Kloas, 2002; Kloas & Lutz, 2006). Based on comprehensive anatomical and morphological data (Duellman & Trueb, 1986; Dumont, 1972; Dumont & Brummett, 1978; Gaupp, 1899; Lofts, 1974; Matthews & Marshall, 1965; Rugh, 1951; Wake & Dickie, 1998; Walker, 1967; Yoshizaki, 1985), these studies primarily focused upon histomorphological and functional effects EDCs caused. No specific attention was paid to the gonadal microvasculature. Dumont (1972) and Dumont and Brummett (1978) who investigated oogenesis in *Xenopus laevis* (Daudin) by light and electron microscopy described the structure of blood vessels within the theca of ovarian follicles and referred to them as “… small capillaries with thin endothelium” (Dumont & Brummett, 1978). Yoshizaki (1985) studied the fine structure of the oviduct epithelium in *Xenopus laevis* but referred only to the pars recta as”…well vascularized….”. Xiang, Burnett, Rawls, Bieber, and Chandler (2004) who focused upon the expression of the sperm chemoattractant “allurin” in Xenopus oviducts only mentioned the presence of a central capillary beneath the mucosal epithelial folds (domes) of the oviduct and of capillaries in between the tubular glands. Due to techniques used, none of these studies presented vascular patterns and spatial relations of ovarian and oviduct vessels.

We here report the three‐dimensional microvascular anatomy of ovary and oviduct in adult *Xenopus laevis* by scanning electron microscopy of vascular corrosion casts in detail (Aharinejad & Lametschwandtner, 1992; Lametschwandtner, Lametschwandtner, & Weiger, 1990; Motta, Murakami, & Fujita, 1992; Murakami, 1971). We could demonstrate that this method is well suited (a) to study the three‐dimensional arrangement of ovarian and oviduct blood vessels, (b) to identify blood vessels as arteries, veins or capillaries (Miodonski, Hodde, & Bakker, 1976, (c) to localize blood flow regulating structures like intimal cushions, muscular sphincters (Aharinejad, Böck, Lametschwandtner, Franz, & Firbas, 1991; Schraufnagel & Patel, 1990), flow dividers, venous valves (Caggiati, Phillips, Lametschwandtner, & Allegra, 2006) and (d) to characterize microvascular patterns within small, yet spatial clearly defined areas within ovaries and oviducts.

## 2 MATERIALS AND METHODS

### 2.1 Animals

Eight adult females of *Xenopus laevis* were studied. Animals (body weight: 18 g–48 g; body lengths: 69 mm–80 mm) were housed in aquaria (tap water depth: 15 cm) equipped with aquarium filters and fed twice a week with either dried *Gammarus pulex* or grinded beef heart.

### 2.2 Histomorphology

Two animals were killed by immersion into an aqueous solution of MS 222 (0.5%). After thoracotomy and exposure of the heart, the venous sinus was opened and the circulatory system was rinsed with Amphibian Ringer solution (Adam & Czihak, 1964) via the arterial trunk (flow rate: 41 ml/hr) and fixed with Bouin`s fixative at the same flow rate. Ovaries and oviducts were excised, rinsed, dehydrated and embedded in paraplast. Transverse and horizontal sections (7 µm) were stained (Goldner) and analysed with an Olympus X51 light microscope. Images were recorded, brightness and contrast of images were adjusted if necessary (Photoshop 7.0; Adobe Inc.). For terminologies we refer to Dumont (1978; ovary) and Yoshizaki (1985; oviduct).

### 2.3 Vascular corrosion casting

Six animals were killed by an overdose of MS 222 (0.5%). For freeing the circulatory system from blood, see 2.2. When clear reflux drained from the opened venous sinus, 10 ml of Mercox CL‐2B (Dainippon Ink and Chemicals; Ladd Burlington) diluted with monomeric methylmethacrylate (4 + 1, v + v, 10 ml monomeric methylacrylate contained 0.85 g initiator paste MA) was injected with a flow rate of 41 ml/hr. When the effluent resin became viscous or the whole amount of resin had been perfused, the injection was stopped. After hardening of the resin, whole animals were tempered, macerated, rinsed and freeze‐dried. Ovaries including oviducts were dissected, mounted onto specimen stubs, sputter‐coated with carbon and gold and examined in the scanning electron microscope XL‐30 (FEI) at an accelerating voltage of 10 kV. For a more detailed procedure of vascular casting, see Lametschwandtner and Minnich (2018).

## 3 RESULTS

### 3.1 Histomorphology

#### 3.1.1 Ovary

**FIGURE 1:**
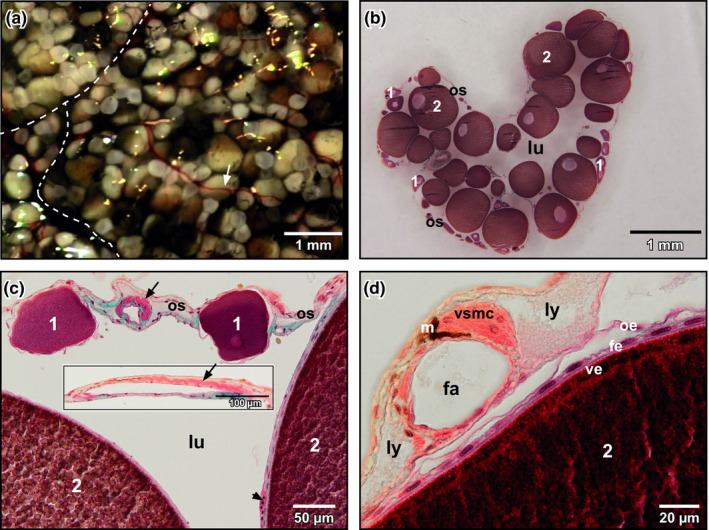
Ovary of adult *Xenopus laevis*. (a) Ventral view. Stereomicroscope image. Dashed lines indicate interlobar borders. Note the different stages of oocytes. Arrow points at an ovarian blood vessel. (b) Ovarian lobe, histomorphology. Tissue section (7 µm), Goldner staining. Light microscope image. Note pre‐vitellogenic (1) and vitellogenic oocytes (2) enclosed by the ovarian sac (os). lu lumen. (c) Interlobar artery. Transverse section (7 µm). Note the horse‐shoe‐shaped bundle of longitudinally arranged vascular smooth muscle cells (arrow). Arrowhead points at a capillary within the follicular theca. Note pre‐vitellogenic (1) and vitellogenic oocytes (2). Inset. Interlobar artery. Longitudinal section (7 µm). Note the bundle of vascular smooth muscle cells (arrow). (d) Follicular artery (fa) and lymphatics (ly). Note the bundle of longitudinally arranged vascular smooth muscle cells (vsmc). fe: follicle epithelium; m: melanocyte; oe: outer ovarian epithelium; ve: vitteline envelope; 2: vitellogenic oocyte

*Xenopus* ovaries consisted of many lobes that contained follicles with pre‐vitellogenic and vitellogenic oocytes (Figure 1a). The ovarian sac, which contained the follicles, consisted of peritoneal epithelium (outer ovarian epithelium), theca and inner ovarian epithelium. The former and the later displayed squamous epithelial cells, the theca connective tissue, fibrocytes and blood vessels. The ovarian lumen extended between follicles (Figure 1b). Interlobar and interfollicular arteries displayed a conspicuous bundle of longitudinally arranged vascular smooth muscle cells (Figure 1c,d). Follicular capillaries located in the theca between inner ovarian epithelium and follicular epithelium (Figure 1c, arrowhead).

#### 3.1.2 Oviduct

**FIGURE 2:**
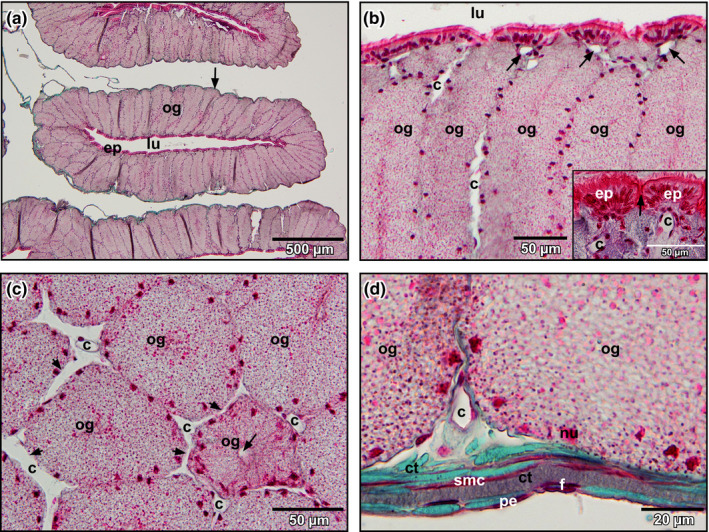
Histomorphology of the oviduct of adult Xenopus. (a) Horizontal section (7 µm) through a segment of the convolute portion (pars convoluta). Note the thin fibrous stroma (arrow). (b) Oviduct epithelium (ep) and oviduct glands (og). Arrows point at subepithelial capillaries (c). Inset. Simple ciliated columnar epithelium forming folds (domes). Arrow points at a glandular opening. (c) Oviduct glands (og). Transverse section. Note that capillaries (c) lying between glands anastomose at different levels (arrowheads). Arrow points at a glandular duct. (d) Fibrous stroma surrounding the oviduct. A capillary (c) locates between the basolateral surfaces of oviduct glands (og). ct: connective tissue; ep: epithelium; f: fibrocyte; lu: lumen; nu: nucleus of glandular cell; pe: peritoneal epithelium; smc: smooth muscle cell

The oviduct pars convoluta consisted (from luminal to abluminal) of a ciliated epithelium, tubular glands below, and a stroma with connective tissue fibres, intermingled fibrocytes, smooth muscle cells and blood vessels (Figure 2a). The ciliated epithelium formed domes with a capillary below (Figure 2b, inset). Between domes, tubular glands opened into the lumen (Figure 2b, inset). Blood vessels (capillaries) located between adjacent glands and anastomosed at different heights with their neighbours (Figure 2c, arrows). Abluminal blood vessels located between the basolateral surfaces of glands and oviduct stroma (Figure 2d).

### 3.2 Vascular anatomy

#### 3.2.1 Ovary vasculature

**FIGURE 3:**
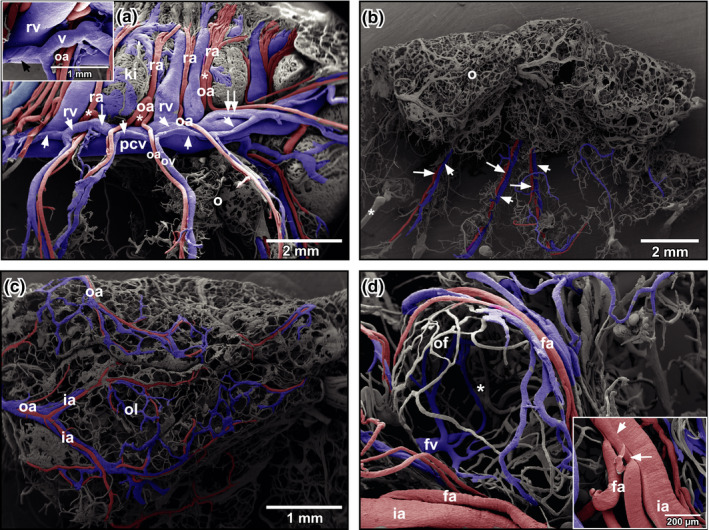
Microvascular anatomy of ovary and oviduct of adult *Xenopus*. Vascular corrosion cast. SEM micrograph. Arteries are coloured red, and veins are coloured blue. (a) Origin of ovarian arteries (oa) from urogenital arteries (asterisks). View at the ventral surface of the left kidney (ki). Right kidney and oviducts are removed. Remnants of the ovary (o) are left. Note the veins (arrowheads) below the posterior caval vein (pcv) which drain several ovarian veins (ov) into the posterior caval vein. Inset. Detail of the vein (v). Note the spirally arranged imprints of vascular smooth muscle cells (arrow). (b) Microvascular anatomy of an isolated ovary. Lateral view. Note supplying ovarian arteries (large arrows) and draining ovarian veins (small arrows) running close aside each other in the mesovarium. Asterisk marks “conductive bridge.”(c) Course and branching pattern of an ovarian artery (oa). Note an interlobar artery (ia) supplying an ovarian lobe (ol). (d) Vascular bed of a single ovoid ovarian follicle (of). Asterisk marks an incomplete filling of the capillary bed. Inset. Intimal cushion (arrow) and flow divider (arrowhead) at the origin of two follicular arteries (fa) from an interlobar artery (ia). fa: follicular artery; fv: follicular vein; rv: renal vein

Urogenital arteries splitted at the ventral surface of the kidneys into renal, ovarian and oviduct arteries (Figure 3a). Ovarian arteries ran within the mesovarium towards the ovary (Figure 3b). In the ovary, they branched into interlobar arteries that ran along the lobar margins (Figure 3c). Interlobar arteries gave off follicular arterioles, which finally fed the capillary network ensheathing ovarian follicles (Figure 3c,d). At the origins of follicular arterioles, intimal cushions were present (Figure 3d, inset). Capillaries formed a wide‐meshed network around ovarian follicles (Figure 3d). After short transition distances, capillaries continued as postcapillary venules, which drained into larger follicular venules (Figure 3d) which gradually merged and became interlobar and finally ovarian veins.

Ovarian veins ran parallel to ovarian arteries (Figure 3a,b). In general, 2–4 ovarian veins first drained into two veins (Figure 3a, arrowheads) which ran ventrally along the posterior caval vein. These veins drained either with an obtuse angle (Figure 3a, arrow) or an acute angle (Figure 3a, double‐arrow) into the posterior caval vein (Figure 3a). They displayed obliquely arranged imprints of vascular smooth muscle cells (Figure 3a, inset).

#### 3.2.2 Oviduct vasculature

**FIGURE 4:**
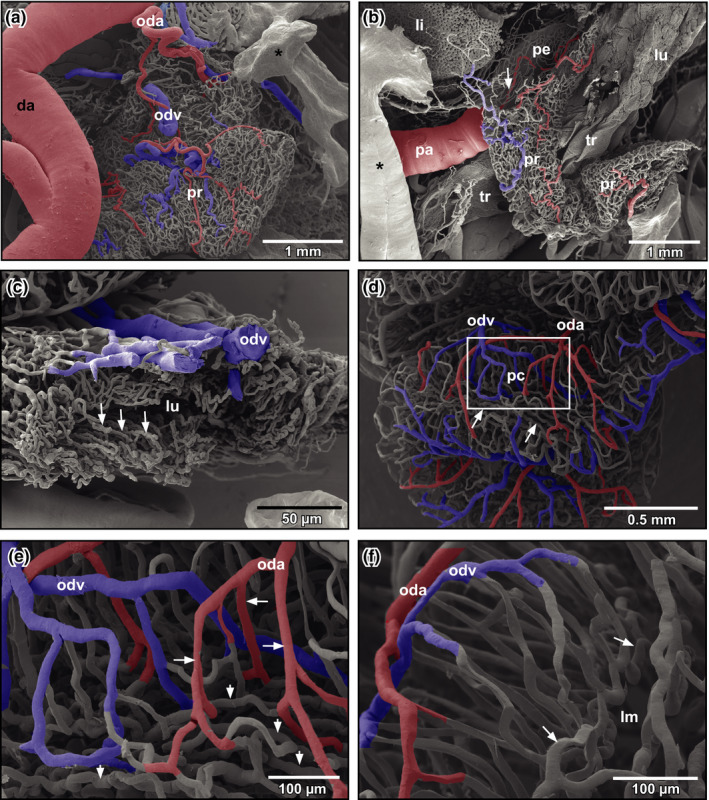
Microvascular anatomy of the oviduct of adult *Xenopus*. (a) Pars recta (pr). Dorsal aspect. Note the first oviduct artery (oda) branching off the dorsal aorta (da). Asterisk marks “conductive bridge.” (b) Microvascuar anatomy of the ostium (arrow) and anterior pars recta (pr) of the oviduct. Dorsal aspect. Note the transition from the capillary network of the slit‐like ostium into the capillary network of the peritoneum (pe). Asterisk marks “conductive bridge.” (c) Microvascular anatomy of the posterior pars recta. Transverse section. Luminal capillary rows (arrows) outline the longitudinally running epithelial folds. (d) Microvascular anatomy of the pars convoluta (pc) of the oviduct. External aspect. Note branching pattern of oviduct arteries (oda), merging patterns of oviduct veins (odv), and wide intercapillary distances. Undulating longitudinally running capillaries can be seen located subepithelially in the depth (arrows). (e) Pars convoluta. Transition of superficially running oviduct arteries (oda) into radially penetrating arterioles and capillaries (arrows). Detail from (d) (enboxed area). Note the wide spacing of blood vessels. Radial capillaries change into longitudinally running undulating capillaries (arrowheads) which drain centrifugally towards superficially located oviduct veins (odv). (f) Pars convoluta. Arteriolar‐capillary‐venular transitions. Slightly oblique longitudinal section. Arrows mark undulating longitudinally running subepithelial capillaries. li: liver; lm: lumen; lu: lung; odv: oviduct vein; pa: pulmonary artery; tr: trachea

The first oviduct arteries branched off the medial aspects of left and right dorsal aortae and supplied ostia and straight portions (pars recta) of oviducts (Figure 4a). Arteries ran towards the ostium and fed the delicate vascular bed of the ostial region, the surrounding two‐dimensional capillary bed of the peritoneum and the remaining portions of the dorso‐ventrally flattened pars recta (Figure 4b,c). The latter displayed luminal capillary loops arranged in longitudinally running rows (Figure 4c, arrows). Few arterioles only supplied the capillary bed. Capillaries drained into postcapillary venules that in turn merged into oviduct veins at the outer layer of the pars recta (Figure 4c).

The convoluted portion (pars convoluta) was highly coiled and had a more cylindric cross section. Oviduct arteries often branched and ran obliquely towards anterior and posterior (Figure 4d). Arterioles and capillaries penetrated the oviduct wall radially (Figure 4e,f). Subepithelially, they changed into undulating longitudinally running capillaries (Figure 4f, arrows), which drained centrifugally towards the outer surface of the oviduct (Figure 4d–f). Oviduct veins finally drained into renal veins or the posterior caval vein.

**FIGURE 5:**
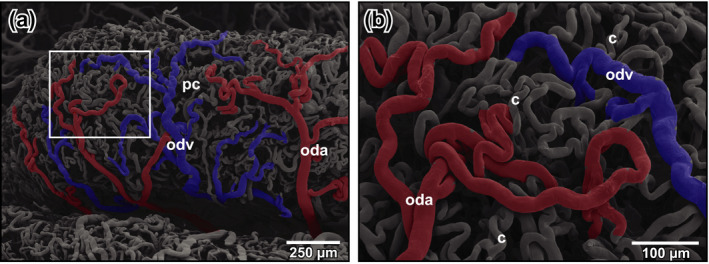
Microvascular anatomy of the pars convoluta in an ovulating adult *Xenopus*. (a) High vascular density with coiled oviduct arteries (oda), undulating oviduct veins (odv) and densely spaced capillaries. (b) Detail from (a) (enboxed area). Arterio‐venous transitions. Note the conspicuous coiling of oviduct arteries (oda), oviduct veins (odv), and capillaries (c)

The density of the oviduct vascular bed differed greatly (compare Figure 4d with Figure 5a,b). While in less densely vascularized specimens, intramural spacing of vessels was wide, that in highly vascularized specimens was very narrow resulting in a close apposition of highly undulating arteries, veins and capillaries (Figure 5a,b).

**FIGURE 6:**
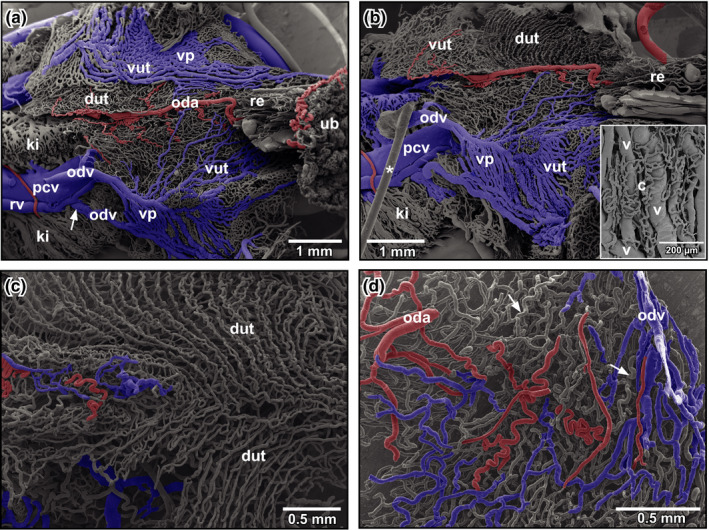
Microvascular anatomy of the uterus (caudal oviduct) in adult *Xenopus*. (a) View at the ventral surface of the ventral (vut) and the dorsal wall (dut) of the uterus . Note the prominent venous plexus (vp) draining into a renal vein (arrow; rv). Part of the ventral wall of the uterus is removed to expose the luminal aspect of the dorsal wall of the uterus (dut). (b) Same as (a), but after removal of the venous plexus of the left uterus. Note the narrow spaced obliquely running capillary rows. Inset. Capillary nets (c) overlaying and draining into veins (v) of the venous plexus. (c) Subepithelial capillary rows of the dorsal walls of right and left uterus (dut). Capillary rows of right and left uterus converge towards the midline and then run longitudinally. (d) Dorsal wall of the uterus. Note supplying arteries (red, oda) and draining veins (blue, odv). Capillary rows at the luminal side displayed in (c) can be seen faintly (arrows). ki: kidney; oda: oviduct artery; odv: oviduct vein; pcv: posterior caval vein; re: rectum; ub urinary bladder

Slightly anterior to the rostral margin of the urinary bladder, the convoluted portions of oviducts became wide, flat and formed uteri (Figure 6a,b). Uteri displayed prominent venous plexuses at their ventral peritoneal surface and drained into the posterior caval vein (Figure 6a,b). Closely attached to the plexuses a network of capillaries was found which originated from and drained into the plexuses (Figure 6b, inset). Luminally, the dorsal walls of uteri displayed capillary rows that extended obliquely from anterior‐lateral to posterior‐medial (Figure 6b,c). At the midline, capillary rows changed directions and ran longitudinally (Figure 6c). Small oviduct arterioles fed the row capillaries (Figure 6d).

## 4 DISCUSSION

In her study on the vascular anatomy of adult *Xenopus laevis,* Millard (1940) performed detailed binocular dissections on gross arterial supply and venous drainage of adult gonads. Due to the limited depth of focus and spatial resolution of the dissecting microscope, she was not able to visualize the microvasculature in detail, but she carefully described and illustrated gross gonadal blood supply and drainage by coloured illustrations Millard (1940). Our results gained by scanning electron microscopy of vascular corrosion casts, a method which also allowed to study the microvascular bed of well circumscribed areas confirmed her binocular findings (Millard, 1940), but allowed also a highly detailed insight into the microvascular anatomy.

In respect to the vasculature of ovarian follicles, we found that vascular profiles embedded in the theca were flat in perfused tissues sections (Figure 3). This finding was confirmed by the flat profiles of cast follicular vessels embracing pre‐vitellogenic and vitellogenic oocytes. These flat vascular profiles most likely reflected the high intravasal resistance that locally resulted in incomplete fillings of the ovarian vascular bed which we found in many of our cast preparations (see Figure 3d, asterisk). Incomplete fillings by their rounded endings were easy to differentiate from broken vessels, which displayed clear sharp endings and from sprouting vessels, which would impose as blind ending gradually tapering structures.

Branching patterns and blood flow regulating structures (intimal cushions, flow dividers) found in our vascular casts reflected the capacity of the ovarian circulation to ensure a sufficient blood supply to oocytes with vital substrates (e.g. vitellin granules synthetized in the liver). The conspicuous intimal cushions found at sites where follicular arterioles branched off interlobar arteries point to the possibility that they regulate blood supply of individual ovarian follicles.

The microvasculature of the convolute portions of the oviduct clearly indicated the ovulatory status of the animal. While the rather loose vascular network most likely reflected the non‐ovulating status, oviducts with dense vascular networks represented the ovulating status. In the latter, intervascular distances down to the capillary bed were extremely small and feeding arterioles often coiled enormously. These findings reflect the intense vascular growth, which ensures an appropriate supply of the highly secretory oviduct glands. These glands deliver the jelly coat to the oocytes when they pass the oviduct.

## CONFLICT OF INTEREST

The authors have no conflict of interests.

## AUTHOR CONTRIBUTIONS

AL performed resin injections, part of processing and SEM analyses of vascular corrosion casts, analyses of tissues sections and figure colorations. AL and BM equally participated in drafting, critical revision and final approval of the manuscript and the figure plates.

## ETHICAL APPROVAL

The study was approved by the Ethics Committee of the University of Salzburg, Austria and the Federal Government (BMBWK‐66.012/0018‐BrGT/2006).

## Data Availability

Further micrographs (LM, SEM) that support the findings of this study are available from the corresponding author upon request.
